# Harmonizing intervention, context, and implementation strategies with Context-Driven Co-Design (CD2)

**DOI:** 10.1186/s43058-026-01020-y

**Published:** 2026-06-25

**Authors:** Sarah A. Birken, Emily R. Haines, Cheyenne R. Wagi, Bonny B. Morris, Kathryn E. Weaver, Kathleen M. Lisborg, Sara J. Landes, Geoffrey M. Curran, Miriam Bender, Maria E. Fernandez, Ashley Strahley, Kimberly Wiseman, Kristie L. Foley

**Affiliations:** 1 Department of Implementation Science, Wake Forest University School of Medicine, Winston-Salem, NC 27101 USA; 2 Cancer Treatment Support, Navigation, American Cancer Society, Atlanta, GA USA; 3 Department of Social Sciences and Health Policy, Wake Forest School of Medicine, Winston-Salem, NC USA; 4 Department of Psychiatry, University of Arkansas for Medical Sciences, Little Rock, AR USA; 5 Behavioral Health Quality Enhancement Research Initiative (QUERI), Central Arkansas Veterans Healthcare System, Little Rock, AR USA; 6 Department of Pharmacy Practice, University of Arkansas for Medical Sciences, Little Rock, AR USA; 7 University at Buffalo, State University of New York, Buffalo, NY USA; 8 Sue and Bill Grossman School of Nursing, University of California, Irvine, CA USA; 9 School of Public Health, University of Texas Health Science Center at Houston, Houston, TX USA

**Keywords:** User-centered design, Context, Implementation, Intervention adaptation, Mid-level managers

## Abstract

**Background:**

Extant approaches to close the gap between implementation evidence and practice have been unsuccessful due in part to overreliance on researcher engagement. In practice, change is often driven by mid-level managers. We developed Context-Driven Co-Design (CD2) to equip mid-level managers with a theory- and evidence-based approach to implementation planning.

**Methods:**

We used a two-pronged design. First, we engaged six established implementation researchers in a modified Delphi process, involving two hour-long meetings and asynchronous feedback to develop and refine a CD2 prototype. Second, we piloted an in-person CD2 training and conducted an hour-long focus group to solicit feedback from mid-level managers on the potential value of CD2 for addressing implementation challenges and opportunities for improving the training. We incorporated focus group feedback and iteratively refined CD2 materials, codified each step of the approach, and further articulated CD2’s key features through two empirical applications.

**Results:**

Delphi panelists unanimously agreed that prototype content would be best delivered through interactive training. The resulting one-time, three-hour in-person training was piloted with 17 mid-level managers affiliated with the National Cancer Institute Community Oncology Research Program who were often tasked with implementing cancer care delivery interventions and research protocols. Focus group participants (*N* = 5) appreciated the training’s practical tools, suggested that the training should focus on a common intervention, and requested more opportunities for peer learning before and after the training. CD2, further refined through subsequent empirical application, involves three steps: (1) agreeing upon an intervention to address a clinical problem and identifying the intervention’s effectiveness-driving features; (2) understanding the context in which the intervention will be implemented, including potential end-users, their workflows, and features of their environment; and (3) co-design session(s) to identify intervention adaptations, context modifications, and exogenous implementation strategies needed to facilitate the intervention’s implementation.

**Conclusions:**

We codified CD2, a theory-driven approach to harmonizing interventions, implementation contexts, and implementation strategies. Mid-level managers found CD2 to be an appropriate and acceptable approach to implementation planning. CD2 is consistent with perspectives that advocate humility and deference of researchers to individuals and contexts that biomedical research often positions as subjects. Future work is needed to enhance CD2’s feasibility and scalability.

**Supplementary Information:**

The online version contains supplementary material available at https://doi.org/10.1186/s43058-026-01020-y.

Contributions to the literature

- Context-Driven Co-Design (CD2) is a structured, theory-driven approach to support healthcare professionals in implementation planning.
- CD2 is based on the principle that organizational inertia can be overcome by leveraging the strategic position of mid-level managers, who can fill structural holes through community-building among key players (e.g., providers, staff, patients, caregivers) to leverage healthcare organizations’ strengths and assets.
- CD2 represents a significant advancement in implementation methodology because it focuses on implementing solutions deemed important by prospective end-users.
- To address problems deemed important by prospective end-users, CD2 acknowledges that any number of interventions and implementation strategies may be useful.

## Introduction/Background

Our 2021 paper “*Harmonizing evidence-based practice*,* implementation context*,* and implementation strategies with user-centered design*” [1] proposed that successful implementation could be achieved through user-centered design, an iterative and highly stakeholder-engaged set of principles, methods [2], and strategies [3]. We presented findings from a pilot study in which we leveraged a subset of user-centered design methods to implement a care coordination intervention for young adults with cancer. Specifically, we applied usability testing to optimize the intervention’s usability and usefulness, ethnographic contextual inquiry to understand the implementation context and identify changes needed to promote receptivity to the intervention’s implementation, and prototyping workshops with a multidisciplinary design team to develop implementation strategies where intervention and context refinements fell short of facilitating implementation.

The approach described in our 2021 paper differs from extant approaches to implementation planning in several ways. First, we intended for our approach to be used by healthcare professionals. This stands in contrast to implementation planning approaches such as implementation mapping, which has been widely used by implementation researchers, but whose complexity may limit use in practice. It also differs from the Systems Analysis and Improvement Approach (SAIA), Designing for Dissemination and Sustainability [4], and the Dynamic Adaptation Process [5], which endeavor to engage healthcare professionals to promote implementation but blur the lines between the roles of researchers and the professionals tasked with the applying the approaches [6–8]. By training healthcare professionals in implementation planning, our approach enhances impact, spread, and scale. Second, unlike approaches that focus on identifying *strategies* to facilitate the implementation of a specific intervention, such as in internal and external facilitation and Collaborative Organizational Approach to Selecting and Tailoring Implementation Strategies (COAST-IS), our approach focuses on the *relationships* among the three pillars of implementation: an intervention, the context into which it is to be implemented, and the strategies intended to facilitate their implementation [9–11]. Third, in contrast to extant implementation planning approaches that have been critiqued as lacking explicit, replicable, consistent descriptions (e.g., facilitation; SAIA), our approach clearly articulates its principles, methods and strategies [12]. User-centered design guides our approach’s methods and strategies. Our approach’s principles are two-fold: (1) Primacy of the priorities of the end-users within a context (e.g., patients, staff, providers) above the objective of intervention implementation. In contrast to many extant implementation planning approaches, ours does not regard implementation as the optimal outcome; an equally optimal outcome may be a decision not to implement. Thus, our approach is consistent with perspectives that advocate for humility and deference of researchers to individuals and contexts that biomedical research often positions as subjects [13]. (2) Overcoming organizational inertia by leveraging the strategic position of mid-level managers. Mid-level managers fill structural holes through community- and relationship-building among key players (e.g., providers, staff, patients, caregivers) to leverage the context’s strengths and assets [14].

In this paper, we report the process and results of a study designed to refine, codify, and pilot our approach for aligning the features of context with an intervention and strategies intended to facilitate implementation. The result, Context-Driven Co-Design (CD2), is a structured, theory-driven approach designed to support healthcare professionals in planning for implementation by integrating state-of-the-science implementation concepts with rich evidence of practice environments’ unique assets and opportunities [15].

## Methods overview

We used a two-pronged design. First, we engaged implementation science experts in a modified Delphi process to develop and refine a CD2 prototype. Second, we piloted and evaluated the prototype with healthcare professionals serving in mid-level manager roles. We report methods and results for each step in turn below. The Wake Forest University School of Medicine Institutional Review Board deemed this study exempt from human subjects review (IRB00101291).

## Modified Delphi panel

### Methods

#### Sampling and recruitment

We leveraged our professional networks and a purposive sampling approach to recruit experts in the three pillars of implementation [1]—interventions, implementation context, and implementation strategies. We sent up to three emails offering potential participants a $250 honorarium to engage in a series of meetings and email communications designed to elicit feedback on iteratively revised versions of the CD2 prototype.

#### Description of processes and materials

We used the study protocol and materials from the study described in Haines et al. (2021) [1] to develop an implementation field guide prototype. The prototype detailed seven steps for harmonizing intervention, context, and implementations strategies: (1) establish a design team, (2) identify intervention’s core functions and forms, (3) understand context, (4) identify friction between intervention and context, (5) adapt intervention, (6) modify context, and (7) address outstanding issues with implementation strategies. Each step included detailed instructions with varying options for carrying out each step depending on resources available, case examples, and resources for carrying out the steps (e.g., interview guide). The initial prototype was then reviewed by an implementation practitioner and revised accordingly.

We convened two hour-long meetings of Delphi panelists via Webex on March 23 and May 6, 2022. Two weeks before each meeting, we sent the implementation field guide, revised between meetings based on previous rounds of feedback, to panelists with requests for written feedback to be discussed during subsequent meetings. Each meeting engaged panelists in open-ended discussions regarding prototype content, format, and proposed delivery. Two investigators took extensive notes and audio-recorded meetings for reference as necessary to inform iterative prototype revisions. After the second meeting, each panelist sent 1–3 rounds of written feedback, according to their preference.

#### Data analysis

Following the first meeting of Delphi panelists, two investigators inductively analyzed meeting notes to identify feedback to be addressed. Based on panelists’ feedback, we abstracted broader cross-cutting themes and iteratively revised the initial field guide to create a CD2 prototype.

### Results

Six implementation scientists participated in the modified Delphi process. Panelists’ institutional affiliations, roles, and areas of expertise are listed in Supplemental File 1. Summaries of feedback themes from the meetings and how feedback was incorporated are listed in Table 1. Briefly, panelists provided feedback on diverse aspects of the prototype, ranging from the mode of delivery to sampling for context assessment interviews. Panelists’ most notable feedback was related to format: they unanimously agreed that the content would be best delivered through an interactive training rather than a field guide. Based on this feedback, we translated the field guide prototype into the CD2 training materials to be delivered to healthcare professionals tasked with translating strategy (i.e., intervention adoption decisions) into action [17].

**Table 1:** Delphi panelists’ feedback

Design Team Feedback	Change Made
**Target audience**: design team encouraged clarification on whether audience was implementation researchers or practitioners	We clarified practitioners as intended audience and focused CD2 content on middle managers.
**Mode of delivery**: design team felt that toolkit would be difficult for busy practitioners to use and digest	We converted the content from a toolkit to an interactive training.
**Novel contribution**: The design team emphasized the importance of documenting how approach differed from existing approaches (e.g., facilitation, implementation mapping)	We reviewed a range of existing methods and found that CD2 is unique in its centering of implementation context in the development of implementation strategies.
**Steps included**: The design team requested clarity around when in the implementation process CD2 should be used, noting that there are no instructions for selecting interventions.	We added introductory content to clarify which stages of intervention implementation are addressed, noting that CD2 is useful once an intervention has already been selected.
**Defining context**: The design team noted that “context” should be further defined and recommended clearly specifying the dimensions of context that CD2 focuses on rather than suggesting that users select their own framework of implementation determinants.	We opted to define context as including three domains: (1) user characteristics and attitudes, (2) workflows, and (3) environment. CD2 still includes flexibility to incorporate implementation frameworks (e.g., using CFIR to select constructs related to environment), but the CD2 context assessment focuses on delving into these three domains of context.
**Design team engagement**: The design team should be engaged earlier in process.	We clarified that the design team is comprised of key informants and other user representatives included in the early stages of CD2.
**Defining process**: More concrete, step-by-step instructions need to be provided for certain steps.	The training articulates step-by-step instructions and includes user-tested materials (e.g., context assessment interview guide) for each phase.
**Sampling**: The target sample size needs to be clarified for data collection activities.	The training emphasizes thematic saturation [16] across user groups for driving context assessment sample size; user group representation to drive number of codesign workshop participants; and consensus to drive number of codesign workshops.
**Actionable friction with context**: There is a need to define what to do with points of friction between intervention and context that are unactionable.	The training acknowledges that not all barriers can be addressed; however, some barriers (or combination of barriers) may suggest aborting the implementation effort to be determined by design team consensus.
**Effort to deploy CD2**: There are concerns about user burden/time constraints, resulting in a need to offer clarification on the effort involved.	We are currently conducting studies of the cost of deploying CD2.
**Post-session activity**: Clarify what happens after co-design sessions.	CD2 is an approach for implementation planning; subsequent steps (i.e., producing context modifications, intervention adaptations, and implementation strategies) have not yet been protocolized.
**Context as a strategy**: Characterize modifying context as an implementation strategy.	The training now describes adapting interventions and developing/selecting implementation strategies, including context modifications.

## CD2 training pilot and focus group

### Methods

#### Sampling and recruitment

We recruited mid-level managers working in the National Cancer Institute Community Oncology Research Program (NCORP) to pilot the CD2 training. NCORP is a network of National Cancer Institute (NCI)-funded institutions that conduct clinical trials and cancer care delivery research in the community, where most cancer patients receive their care [18]. Participants were recruited through multiple notices in the Wake Forest NCORP Research Base Study Matters biweekly newsletter, which is distributed to a listserv of over 6,600 contacts including NCORP research and clinical staff, such as cancer care delivery research (CCDR) leads (i.e., mid-level managers), who facilitate the implementation of cancer care delivery protocols and interventions in NCORP community sites. CCDR leads who were interested were asked to complete a REDCap form to register for the in-person training, and reminders were sent with further details.

#### Description of processes and materials

We piloted the in-person CD2 training on October 18, 2023, the day after the Wake Forest NCORP Research Base annual meeting in Asheville, North Carolina, USA. The training engaged participants in a discussion regarding implementation challenges in practice; provided an overview of implementation science and user-centered design; and then guided participants through the CD2 approach, including adapting interventions, modifying implementation contexts, and co-designing implementation strategies. We iterated between didactic sessions and small-group discussions on each topic. Each participant received the materials including an agenda, slides on didactic sessions, CD2 data collection instruments, tips for co-design sessions, a list of implementation strategies [19], and a glossary of referenced terms.

An experienced mixed methods researcher on our study team engaged a subset of training participants who were willing and able to participate in a single hour-long semi-structured focus group directly following the CD2 training to solicit input on the training as well as the CD2 approach itself. The focus group guide can be found in Supplemental File 2. The focus group was audio-recorded and transcribed verbatim.

#### Data analysis

Two experienced qualitative researchers from Atrium Health Wake Forest Baptist Comprehensive Cancer Center’s Qualitative and Patient-Reported Outcomes shared resource reviewed the focus group transcript to inductively develop a codebook based on emergent themes related to feedback on the features of the CD2 training’s content and delivery. Data were managed using ATLAS.ti version 23. The two researchers independently coded the focus group transcript and met to compare coding and resolve discrepancies. Segments of text were reviewed by code and summarized. Summaries were then synthesized into themes using the principles of thematic analysis [20].

### Results

In total, 17 registrants participated in the CD2 training, representing the NCI and eight separate NCORP community oncology research Sites. From the NCORPs, seven participants identified themselves as CCDR Leads, five identified as an Administrator/Director/Manager, two were Investigators, and one represented Biostatistics. CCDR leads represented six states- Illinois, South Carolina, Missouri, Georgia, Minnesota, and Pennsylvania.

Five CCDR leads representing seven NCORP community sites consented to participate in the focus group. CCDR leads provided feedback on the CD2 approach and training (see Table 2). Specific suggestions made during the focus group included: incorporating into the CD2 materials a “one-pager” on the intervention of focus to share with stakeholders engaged during CD2; adding more content to the CD2 training on best practices for data collection to equip trainees with the skills they need to complete the context assessment; incorporating role playing into the CD2 training to prepare trainees to facilitate co-design sessions; focusing the training on a common intervention; and providing opportunities for healthcare professionals delivering CD2 to connect and share experiences.

**Table 2:** CD2 participant feedback

Topic	Feedback
Lack of clarity around purpose of the training and suggested communicating the purpose and required preparation with participants in advance	“I think maybe if there was more intentional communication, like come to this meeting with something you want to implement.”
Participants reported appreciating the materials they received	“I think for me, the tools, again, were extremely valuable.”
Having tools in advance would help them work on their idea/challenge	“Maybe some of the tools ahead of time too. You’ve got your challenge, but you’re workin’ through your flow because if you get here and you see that tool for the first time and go, “Oh, yeah. This is really helpful,” you’ve still gotta be thinking about, okay. I’m using this tool, and who would that be, and this or that.”
The introduction to implementation science was a useful piece of the training	“I think an introduction to the background of implementation science is really interesting and showing how you can help get things done, so I guess that goes back to about the theory. Do you wanna know about the theory? Part of me does, and part of me doesn’t, but [laughter] I do think it’s helpful.”
Participants enjoyed the opportunity to work with other CCDR leads	“I loved the ability to collaborate with people from different NCORPs and hear about the things that are weighing on them that they are trying to implement.”
Participants felt there was a benefit to discussing challenges and sharing ideas	“That’s why I like going to these meetings to hear, okay. What’s successful? How are you successful there versus here? What are you doing differently? How can I adopt whatever it is that you’re doing at my institution?”
Participants felt ongoing engagement would be beneficial	“[…] doing something quarterly—How did you guys do? What did you learn? What were your failing points? How can we help you?”
One participant said some of the language, such as “stakeholder” was unfamiliar	“Not to sound silly, but as a research nurse […] when they said stakeholders, I’m like, ‘I don’t even know what they’re talkin’ about.’ The language and understanding what that language means—‘cause I’m like, ‘Oop, they’re way over my head. I don’t even know what they’re talkin’ about.’”
One participant commented the training was not long enough	“a little short.”
Lack of clarity around purpose of the training and suggested communicating the purpose and required preparation with participants in advance	“I think maybe if there was more intentional communication, like come to this meeting with something you want to implement.”
Participants reported appreciating the materials they received	“I think for me, the tools, again, were extremely valuable.”
Having tools in advance would help them work on their idea/challenge	“Maybe some of the tools ahead of time too. You’ve got your challenge, but you’re workin’ through your flow because if you get here and you see that tool for the first time and go, “Oh, yeah. This is really helpful,” you’ve still gotta be thinking about, okay. I’m using this tool, and who would that be, and this or that.”
The introduction to implementation science was a useful piece of the training	“I think an introduction to the background of implementation science is really interesting and showing how you can help get things done, so I guess that goes back to about the theory. Do you wanna know about the theory? Part of me does, and part of me doesn’t, but [laughter] I do think it’s helpful.”

## CD2 codification

### Methods

Two investigators incorporated feedback from the focus group into the CD2 approach, training, and materials. Over the course of two years, through two applications of CD2 related to implementing social needs screening in diverse cancer care contexts [21, 22], the two investigators further iteratively refined all CD2 materials, codified each step of the approach, and further articulated CD2’s key features. Descriptions and results of these two applications will be reported elsewhere; here, we focus on presenting the CD2 approach that culminated from this body of work.

### Results

Our CD2 development process culminated in a three-step approach for implementation planning and an accompanying training to equip mid-level managers with the skills needed to facilitate CD2. Table 3 describes CD2 steps as well as key features and considerations for each step. Supplemental File 3 includes the CD2 Key Informant Interview Guide, and Supplemental File 4 includes the CD2 Observation Template. The interview guide and observation template are intended to be used semi-structurally, with flexible ordering and probing to elicit evidence regarding a wide array of contextual influences.

**Table 3:** Context-driven co-design steps

Step	Feature	Rationale
Understand the intervention	Focus on an intervention’s core functions rather than forms [39]	Allows for flexibility across contexts in terms of HOW the goals of an intervention can be achieved, accounting for variation in staffing, workflows, and other key contextual features
	Overarching focus on usability	Acknowledges usability as a determinant of perceptual implementation outcomes (feasibility, acceptability, etc.) which, in turn, influence behavioral implementation outcomes (adoption, reach)
Understand the context	Identification of one individual to serve as key informant. This individual should have capability (knowledge of context and potential to be an agent of change), opportunity (bandwidth to engage in change effort), and motivation (intrinsic interest in engaging in change effort) [22]	Key informant acts as a connector to other relevant user groups and other important considerations; provides access to the context, facilitating participation and buy-in from other users; and leads the CD2 process, or works closely with CD2 facilitator throughout, to ensure that their unique context remains the focal point
	Interview with key informant	In this interview(s), the key informant provides a broad snapshot of the context and the status of intervention implementation. During the interview, the key informant also indicates important user groups to engage during clinic observation and other important considerations
	Clinic observation with other key user groups	Clinic observation provides an opportunity to conduct “miniature interviews” with multiple individuals working within a context while they work, bypassing the logistical complexity and burden of scheduling separate interviews. Observation also provides information beyond what could be gleaned in interviews on workflows (what users DO versus what they SAY; how users flow through a clinic), and features of the physical environment
Co-design intervention adaptations, context modifications, and exogenous implementation strategies	Inclusion of different user groups (e.g., providers, staff, patients) in the same co-design session(s); rapport-building and expectation-setting to mitigate potential power imbalances	Allows for triangulation across perspectives and consensus-building; addressing power imbalances can help encourage candid participation from all parties; creates a community around the implementation effort
	Additional vetting of intervention and context findings by design team	Ensures accurate interpretation of contextual data
	Visual and interactive representations of workflows and other contextual information to design team	Enhances design team participation and results in rich feedback to undergird implementation plan
	Prioritizing making the intervention as usable and the context as receptive to implementation as possible before considering exogenous implementation strategies	Minimizes burden associated with executing implementation plan
	In situations where implementation is not mandated, allowing for the decision not to implement to be an acceptable outcome of CD2	Prioritizes researchers’ humility and acknowledges that the people operating within a context have more knowledge of the needs and constraints of that context than external parties (e.g., researchers); acknowledges that the successful implementation and sustainment of an intervention relies on buy-in from change agents

#### Step one

This step involves working with individuals within a context (i.e., potential end-users) to reach consensus on the clinical problem of focus and the proposed solution to the problem (“the **intervention**”). Once the intervention is selected, its known or suspected core functions (i.e., effectiveness-driving features in terms of goals that the features accomplish and/or specific activities required to accomplish goals) must be identified. Methods of identifying core functions are based on those developed by Kirk et al. [23] Once core functions are identified, CD2 includes engaging potential end users to review how the intervention’s core functions have been achieved in other contexts (i.e., the intervention’s forms in other contexts) and comment on their potential usability (i.e., ease of use) in the new context.

#### Step two

The second step is understanding the **context** in which the intervention will be implemented, including potential end users, their workflows, and features of their environment. This step includes conducting a context assessment comprised of two parts, each guided by a CD2 data collection form: (1) interview(s) with one (or more) key informant(s) to provide an overarching understanding of a context and other key users groups to engage within that context (see Supplemental File 3), and (2) clinic observation and interaction with other key user groups (e.g., informal interviews) to explore perspectives beyond that of the key informant and to understand workflows and other processes that will impact the intervention’s implementation (see Supplemental File 4). We have used the Maguire et al. framework to guide CD2 context assessment; this framework from user-centered design conceptualizes context in terms of user characteristics and attitudes, their workflows, and their environment [2]. Other implementation determinant frameworks may be used; however, we selected a framework that divides context into three broad domains that we believed would be intuitive to healthcare professionals; preliminary findings suggest the usability of this simple contextual framework among those without extensive knowledge of implementation science concepts.

#### Step three

The third step is engaging a design team comprised of prospective end users (e.g., patients, clinicians, staff) and other key individuals in co-design session(s) to review data on intervention and context, identify important points of friction, and generate prototypes of **intervention adaptations**,** context modifications**,** and any exogenous implementation strategies** needed to facilitate the intervention’s implementation.

## Discussion

We developed, piloted, and codified CD2, an innovative approach to implementation planning in healthcare settings. Our modified Delphi panel resulted in a three-hour CD2 training that we piloted with mid-level managers who were tasked with implementing research protocols and interventions. Mid-level managers indicated that CD2 was an appropriate and acceptable approach to implementation planning and provided valuable input for refinement of the approach in future work. Thus, CD2 represents a significant advancement in implementation methodology by codifying a theory-driven approach to enhance reproducibility in real world settings and overcoming extant implementation approaches’ challenges with uptake in practice [24]. The process of codifying CD2 clarified its underlying principles, including strengths- and assets-based implementation, community-building, and organizational change. Organizations tend toward inertia, impeding change associated with implementing interventions [25]. Organizational inertia can be overcome in part by leveraging the strategic position of mid-level managers, who fill structural holes in healthcare organizations [14] Importantly, CD2 focuses on implementing solutions deemed important by prospective end-users rather than any specific intervention or implementation strategies. To address problems, CD2 acknowledges that many interventions and implementation strategies may be used [26]. Central to CD2 is understanding relationships among the three pillars of implementation (i.e., intervention, context, and implementation strategies) and leveraging relationships among end-users within healthcare organizations (e.g., patients, staff, providers) to understand and modify interventions and contexts to support implementation. The critical importance of relationships among the three pillars of implementation is consistent with extant evidence regarding implementation strategies: A review of 86 reviews of implementation strategies concluded that future research should “shift its focus from merely examining ‘what works’ to ‘what works where and what works for whom.’” [27] The relational centerpiece of CD2 is also consistent with the extant evidence regarding implementation strategies [28]: Many of the strategies for which there is some evidence of improving implementation involve building relationships among implementation partners (e.g., facilitators, providers, staff) [29]. For example, in their mechanistic analysis of practice facilitation to promote the reach of an evidence-based secure firearm storage program in pediatric primary care, Williams et al. [29] did not find support for their hypothesis that the relationship was mediated by practice adaptive reserve (i.e., capacity for successful change management [30]); rather, the team’s qualitative findings suggested that the effect was driven in part by the relationships fostered between facilitators and providers. This relational focus is consistent with the theory that trusting relationships improve motivation, opportunity, and capability among implementation partners and, in turn, implementation outcomes [31]. Our findings offer an approach to support teamwork to facilitate implementation, a critical topic that has received scant attention in the field and respond to calls for empirical evidence of implementation tailoring in action [8, 32].

CD2 overcomes the limitations of many established implementation planning approaches because we designed it to (1) be used by healthcare professionals (i.e., mid-level managers); (2) focus on relationships among the three pillars of implementation (i.e., context, intervention, and implementation strategies); and (3) specify reproducible steps that may be tailored to meet users’ needs. Conceptually, CD2 is consistent with Participatory Implementation Systems Mapping (PISM) in a shared emphasis on the community-building that occurs as a group ideates around a shared concern and a conceptualization of resistance to change as a feature of system rather than a barrier to be overcome. However, CD2 differs from PISM in some key respects. First, while PISM might be reasonably applied in a clinical context, the case example offered relates to a problem of much greater scale (i.e., chronic disease prevention). In contrast, CD2 is meant to address a discrete problem in a specific clinical context. Second, PISM’s system-building function requires large amounts and types of data that may be inaccessible or impractical from the standpoint of the healthcare professionals we intend to use CD2. CD2 is in active use in diverse settings. For example, we received funding from the NCI to pilot CD2 in our comprehensive cancer center’s supportive care clinic to facilitate the implementation of the Epic Social Determinants of Health Wheel, a standardized social drivers of health screening tool. The resulting compilation of co-designed strategies, CareConnect, increased the reach of social drivers of health screening from 38.4% to 78.8% of patients. Further, CD2 participants, including supportive care clinic providers, staff, and patients, reported that CD2 was an acceptable, appropriate, and feasible approach to implementation planning because it aligned with clinical workflows and priorities. CD2 is also currently being tested in community oncology clinics across the United States to enhance social needs screening. Thus, CD2 shows promise for overcoming extant implementation approaches’ challenges with uptake in practice [24].

CD2 and our methods of developing and piloting the approach have limitations. CD2 was designed to be intervention-agnostic; however, to date, we have largely applied CD2 to needs screening interventions (e.g., health-related social needs). To optimize CD2, we must assess its effectiveness in facilitating the implementation of diverse interventions. Mid-level managers included in our pilot were limited to those engaged in NCORP CCDR; however, they represented diverse roles in implementation practice, including clinical and research staff and providers. Although CD2 is designed for administration by mid-level managers, to date, trained implementation scientists have partnered with mid-level managers in CD2 studies, limiting its scalability. The specific skills and characteristics required of CD2 facilitators remain unclear. We will shed light on required skills and characteristics of CD2 facilitators in our currently funded NCORP project in which we are training a broad range of mid-level managers (e.g., nurse managers, CCDR leads). Future work is needed to promote CD2’s scalability by estimating the cost of deploying CD2.

Current and planned efforts are specifically designed to address these limitations. For example, our currently funded work explores the feasibility of training NCORP practice staff to administer CD2 in preparation for a subsequent trial in which we plan to test CD2 against generalized (i.e., not context-specific) implementation guidance and usual care (i.e., no implementation guidance). We are also pursuing opportunities to translate the CD2 training into a longitudinal training with learning collaboratives, perhaps leveraging extant infrastructure such as Project ECHO, in which practitioners are engaged in a virtual learning community. In addition, to address the significant time, human, and financial resource demands that limit CD2’s scalability, when certain features of an implementation effort are held constant (e.g., rapid adaptation of CD2-derived implementation strategies from one context to meet the needs of another similar context seeking to implement the same intervention), we are exploring opportunities to condense the CD2 process.

We present this paper to codify CD2 and facilitate empirical testing of the approach, which holds promise for practice and policy. Various accrediting bodies and professional organizations establish standards that healthcare organizations struggle to implement [33–35], and rapidly consolidating health systems are often challenged to standardize policies and procedures [36, 37]. CD2 offers guidance for implementing standards in ways that attend to local conditions, addressing lack of buy-in from key interest-holders and leveraging evidence-based strategies for overcoming resistance to change, such as collaborative decision-making and transparent communication. Thus, CD2 represents a structured approach to facilitating implementation that could complement existing standards for implementation facilitators [38].

## Conclusions

CD2 represents a promising approach to facilitating implementation, operationalizing the theoretical foundations described in our previous work. Through several coordinated projects, we generated preliminary evidence of CD2’s acceptability and appropriateness. CD2 addresses the limitations of many extant implementation planning approaches, in part by engaging mid-level managers, who are in an ideal position to foster the relationships required to harmonize interventions, contexts, and implementation strategies to support implementation. CD2 holds promise for policy and practice. Future work is needed to enhance CD2’s feasibility and scalability.

## Supplementary Information

Below is the link to the electronic supplementary material.

Supplementary Material 1

Supplementary Material 2

Supplementary Material 3

Supplementary Material 4

## Data Availability

Datasets used and/or analyzed during this study are available from the corresponding author on reasonable request.
