# Supplementary material for: Harmonizing intervention, context, and implementation strategies with Context-Driven Co-Design (CD2)
Source: Implement Sci Commun. 2026 Jun 25;7:184. doi: 10.1186/s43058-026-01020-y (PMC13560442; doi:10.1186/s43058-026-01020-y)
Supplement: Supplementary file 1 [file 43058_2026_1020_MOESM1_ESM.pdf]

Supplemental File 3. Focus Group Interview Guide

NCORP Annual Meeting

CD2 Workshop Evaluation Focus Group

Thank you for agreeing to speak with us today. The purpose of this focus group is to obtain feedback on the CD2 workshop and suggestions on how we can train people on CD2 based on your experience in this workshop. Our discussion should last about 1 hour. All of your responses will remain confidential within this focus group. You may choose to leave the focus group at any time, and there is no penalty to you for not completing the meeting.

Your identity will be known to other focus group participants and the researcher(s) cannot guarantee that others in the group will respect the confidentiality of the group. As a researcher, we ask that you keep all comments made during the focus group confidential and not discuss what happened during the focus group outside the meeting.

(If participant is a WAKE FOREST employee, add: Taking part in this research is not a part of your University duties, and refusing will not affect your job. You will not be offered or receive any special job-related consideration if you take part in this research.)

Before we begin, we would like to ask your permission to audio record our discussion. Would it be OK with you if I record our discussion? The recording will be used for training and transcription purposes. The focus group will be transcribed, however, your name or any personal identifiers will not be associated with any of the notes. The audio recordings will be deleted once the project is complete. If you have any questions, you can reach out to Emily Haines, the PI [ehaines@wakehealth.edu].

There are two purposes of this focus group: First, we'd like your feedback on the CD2 approach itself—whether you see value in it or parts of it, the feasibility of doing it in your practice, etc. Second, we'd like feedback on how we can train people in using CD2 moving forward based on your experience in this workshop.

I'd like to begin by hearing your thoughts on CD2 as an approach to facilitating implementation.

- In your experience, what have been the primary barriers or issues that have come up as you've tried to implement evidence-based interventions?
- What do you see as the value of CD2 as an approach to implementation?
- What are its limitations?
- Did the information presented about CD2 make sense? What did/didn't make sense?
- How does CD2 compare to other efforts to facilitate implementation of evidence-based practices (e.g., QI collaboratives)?
- To what extent is it feasible for you to do the steps that CD2 requires: doing context assessment; recruiting a codesign team; conducting codesign sessions?

Now I'd like to shift to hearing your feedback on this workshop as a means of helping you to use CD2.

- To what extent will this workshop help you to use CD2 for implementing interventions such as HRSN screening?
- What else do you need to support you in using CD2 for HRSN screening implementation? What about future interventions?
- What did you like about the workshop? What didn't you like? E.g., amount of time; topics discussed; lecture vs. breakouts; Emily and Sarah; characteristics of others in the group (e.g., same/different problems they're facing)
- We recognize that this workshop alone is probably insufficient to facilitate the highest quality implementation. E.g., you go back to your office and are overwhelmed/don't follow up. What else would you need to move forward with CD2? (e.g., technical assistance; academic detailing; regular peer-to-peer meetings)
