# Supplementary material for: Harmonizing intervention, context, and implementation strategies with Context-Driven Co-Design (CD2)
Source: Implement Sci Commun. 2026 Jun 25;7:184. doi: 10.1186/s43058-026-01020-y (PMC13560442; doi:10.1186/s43058-026-01020-y)
Supplement: Supplementary file 2 [file 43058_2026_1020_MOESM2_ESM.pdf]

Supplemental File 1. Delphi Panelists

Panelist Name	Institutional Affiliation	Roles	Areas of Expertise	Relevant Expertise for CD2
**Miriam Bender, PhD, MSN**	UC Irvine Sue & Bill Gross School of Nursing	**Associate Professor,** Sue & Bill Gross School of Nursing<br><br>**Founding Director**, Center for Nursing Philosophy	Clinical Leadership, Healthcare Processes and Outcomes, Nursing Care Models, Nursing Philosophy, Organizational Theory	Context assessment
**Geoffrey Curran, PhD, MA**	University of Arkansas for Medical Services College of Pharmacy	**Professor,** Pharmacy Practice	Implementation Science, Diffusion of Innovation, Quality Improvement	Hybrid designs
**Laura Damschroder, MS, MPH**	Center for Technology and Behavioral Health	**Sole Proprietor,** Implementation Pathways, LLC<br>**Research Investigator**, Veterans Affairs (VA) Ann Arbor Center for Clinical Management Research **Principal Investigator**, Maintaining Implementation through Dynamic Adaptations (MIDAS) QUERI program	Consolidated Framework for Implementation Research (CFIR), Healthcare Systems, Veteran Health, Evidence-based Practice Translation	Context assessment
**María Fernández, PhD**	University of Texas Health Science Center at Houston	**Vice President**, Population Health & Implementation Science<br>**Lorne Bain Distinguished Professor of Public Health & Medicine**<br>**Professor**, Department of Health Promotion & Behavioral Sciences	Dissemination & Implementation, Community-based Participatory Research, Health Equity, Global Health, Cancer Prevention & Control, Non-communicable Diseases	Intervention design

		**Director**, Center for Health Promotion & Prevention Research		
**Sara Landes, PhD**	University of Arkansas for Medical Services Psychiatric Research Institute	**Associate Professor**, Department of Psychiatry<br>**Clinical Psychologist**, Central Arkansas Veterans Healthcare System<br>**Director,** Behavioral Health Quality Enhancement Research Initiative (QUERI) Program<br>**Investigator**, VISN 16 MIRECC, Central Arkansas Veterans Healthcare System<br>**Research Health Scientist**, Center for Mental Healthcare and Outcomes Research, Central Arkansas Veterans Healthcare System<br>**Core Faculty**, Center for Implementation Research, University of Arkansas for Medical Sciences	Evidence-based Treatment Implementation, Mental and Behavioral Health, Suicide Prevention, Dialectical Behavior Therapy (DBT), Healthcare Systems, Veteran Health	Hybrid designs
**Bryan Weiner, PhD**	Department of Global Health and Health Services at the University of Washington	**Professor**, Department of Global Health and Department of Health Services	Implementation Science, Evidence-based Practices in Healthcare Delivery, Qualitative Research and Methods, Global Health, Cancer, Non-communicable disease	Context assessment; implementation strategies
