# Supplementary material for: Harmonizing intervention, context, and implementation strategies with Context-Driven Co-Design (CD2)
Source: Implement Sci Commun. 2026 Jun 25;7:184. doi: 10.1186/s43058-026-01020-y (PMC13560442; doi:10.1186/s43058-026-01020-y)
Supplement: Supplementary file 3 [file 43058_2026_1020_MOESM3_ESM.pdf]

CD2 Key Informant Interview Guide

This interview guide was designed to be broadly applicable to a wide range of projects using CD2. Thus, the interview guide should be tailored to your project, including the specific intervention (or problem for which an intervention is being sought); the purpose of the project (e.g., initiating, improving, or replacing an implementation process); and the relationship between the interviewer and the interview participant (e.g., established working relationship vs. external facilitator). Guidance for using this observation template is available in the CD2 training.

Introduction

Thank you for agreeing to speak with me today. The purpose of this interview is to understand current practices in addressing [insert clinical problem] in [insert clinical setting]. Our discussion should last about 45 minutes. All of your responses will remain confidential within this study. You may choose to stop this interview at any time, and there is no penalty to you for not completing the interview.

We will keep your identity confidential, making every effort to ensure that your specific responses are not attributable to you, although we may report findings from this interview in aggregate along with findings from interviews with others.

Taking part in this interview is not a part of your duties at [institution], and refusing will not affect your job. You will not be offered or receive any special job-related consideration if you take part in this interview.

Before we begin, we would like to ask your permission to audio record our discussion. Would it be OK with you if I record our discussion? The interview will be transcribed; however, your name or any personal identifiers will not be associated with any of the notes. The audio recordings will be deleted once the project is complete. If you have any questions, you can reach out to [insert project lead's name and contact information].

Key Informant characteristics

1. What is your role in [insert clinical setting]?

Overall status of implementation

2. Is [insert clinical problem] being addressed in your setting through [insert intervention] or any other efforts?

Broad snapshot of current intervention use or related processes

IF 2=yes

3. What is your role in the intervention/related processes?
4. Can you walk me through the intervention/related processes?

a. Who is involved?
   i. Who administers the intervention?
   ii. Who receives the intervention?
b. Describe workflows related to intervention/related processes.
   i. Where do they occur?
   ii. When? At what frequency?

IF 2=no

5. How do you think the [insert clinical problem] could be addressed in your setting?
6. How might the intervention selected to address the [insert clinical problem] be implemented in your setting?
   a. Who would be involved?
      i. Who would administer the intervention?
      ii. Who would receive the intervention?
      iii. Whose buy-in would be needed to implement the intervention?
   b. How might the intervention be delivered in light of existing workflows?

Implementation determinants

7. What has been/would be helpful to implement intervention?
   a. ...in terms of the people in your setting- their attitudes, characteristics?
   b. ...in terms of workflows and processes in your setting?
   c. ...in terms of the environment (organizational, social, technical, physical, broader community, political)?

8. What has stood/will stand in the way of implementing the intervention?
   a. ...in terms of the people in your setting- their attitudes, characteristics?
9. ...in terms of workflows and processes in your setting?
10. ...in terms of the environment (organizational, social, technical, physical, broader community, political)?

Next steps

Next, I will spend some time doing observation in [insert clinical setting]. I would like some input from you to help make the best use of that time.

11. Who are the people that I really need to talk to about this intervention or its implementation in your setting
12. Who are the three people I should prioritize for scheduling time with?
13. What should I consider in terms of scheduling observation session (days of the week, clinic times, who is on the schedule and when, etc.)?
14. What should I make sure I pay attention to (e.g., specific processes, aspects of the physical environment) when I go in for clinic observation?
