# Supplementary material for: Harmonizing intervention, context, and implementation strategies with Context-Driven Co-Design (CD2)
Source: Implement Sci Commun. 2026 Jun 25;7:184. doi: 10.1186/s43058-026-01020-y (PMC13560442; doi:10.1186/s43058-026-01020-y)
Supplement: Supplementary file 4 [file 43058_2026_1020_MOESM4_ESM.pdf]

CD2 Clinic Observation Template

This observation template was designed to be broadly applicable to a wide range of projects using CD2. Thus, the observation template should be tailored to your project, including the specific intervention (or problem for which an intervention is being sought); the purpose of the project (e.g., initiating, improving, or replacing an implementation process); and the relationship between the observer and the observed (e.g., established working relationship vs. external facilitator). Here, we have used health-related social needs (HRSN) to provide examples of each domain; these should be replaced with project-specific information. Guidance for using this observation template is available in the CD2 training.

User's Role: ______________________
(e.g., patient, social worker, oncologist, etc.)

Users	Workflows	Environment
Characteristics and attitudes of user.	Descriptions of actions/ work performed by user.	Descriptions of any features of social, technical, organizational, or physical environment.
Example: "social worker says they think HRSN screening is extremely important. They are aware of the Epic Wheel but have no experience using it. They seem to have a strong understanding of services and resources available in-house and in community"	*Example: "social worker conducts initial consultation with most, but not all new clinic patients. These visits may occur in-person or virtually. During these consultations, they assess HRSN but through an informal/conversational approach. They record information from these conversations in a clinic note."*	*Example: "social worker says they often meet with patients in the infusion space, which lacks privacy and thus is not ideal for more sensitive or private conversations."*<br>*"social worker notes that clinic leaders are very supportive of efforts related to health equity and social needs"*<br>*"clinic has iPads available in the waiting room which patients use to complete intake forms"*

Context-Driven Co-Design
